# Impact of sleeve gastrectomy compared to Roux-en-y gastric bypass upon hedonic hunger and the relationship to post-operative weight loss

**DOI:** 10.1007/s11739-022-03063-0

**Published:** 2022-08-14

**Authors:** Janine Maria Makaronidis, Andrea Pucci, Marco Adamo, Andrew Jenkinson, Mohamed Elkalaawy, Rachel Louise Batterham

**Affiliations:** 1 Division of Medicine, UCL Centre for Obesity Research, University College London, London, UK; 2 Bariatric Centre for Weight Management and Metabolic Surgery, University College London Hospital, London, UK; 3 National Institute of Health Research, UCLH Biomedical Research Centre, London, UK

**Keywords:** Obesity, Bariatric surgery, Power of food, Sleeve gastrectomy, Hedonic hunger, Weight loss

## Abstract

‘Hedonic hunger’ indicates the desire to consume food in the absence of an energy requirement. Hedonic hunger can be investigated using the validated Power of Food Scale (PFS). Roux-en-Y gastric bypass (RYGB) and sleeve gastrectomy (SG) are currently the most effective treatment options for severe obesity. Following RYGB, hedonic hunger diminishes, which may contribute to sustained weight loss. There are no data examining the effect of SG on hedonic hunger. We prospectively evaluated hedonic hunger using PFS in patients with severe obesity prior to and 6 months after SG (*n* = 95) or RYGB (*n* = 44) and investigated the procedure-specific relationship between percentage weight loss (%WL) and hedonic hunger. Anthropometric data were collected at baseline after 6 months, 12 months and 24 months post-operatively. PFS contains 15 items grouped into 3 domains considering when food is: available (FA), present (FP), tasted (FT) and a total score (TS). At 6 months, a significant reduction was seen in all categories post-SG (*p* < 0.0001) and in TS (*p* = 0.003), FA (*p* = 0.0006) and FP (*p* = 0.0007) post-RYGB. A significantly larger reduction in FP scores was seen post-SG (*p* = 0.01). Post-SG, a significant correlation with 6-month %WL was noted for changes in FP (*p *= 0.03) and TS (*p* = 0.03). Post-SG changes in FP and TS predicted 24-month %WL. Post-RYGB significant correlations were seen between 6-month %WL and dFA (*p* = 0.04) and dFP (*p* = 0.03). Changes in FA, FP and TS were predictive of 12-month %WL. HH is reduced following both SG and RYGB with a greater reduction following SG and is related to post-operative %WL. PFS may have a role as a predictive tool for post-operative outcomes following SG and RYGB.

## Introduction

During the majority of human history, the primary driver for seeking food has been survival. However, eating constitutes a behavior, and in the modern environment, the average person is faced with over 200 food-related decisions daily. Food-related decision-making therefore goes far beyond meeting energy requirements [1]. The phenomenon where reward drives eating in the absence of an energy requirement is termed “hedonic hunger” (HH) [2]. The widespread availability of energy-dense and palatable foods can be a major contributor to weight gain and obesity; and is thereby closely linked to the rising prevalence of metabolic conditions, including type 2 diabetes (T2D) and cardiovascular disease [3, 4]. Obesity is associated with a significant reduction in life expectancy but also has a detrimental impact upon quality of life.

Body weight is regulated by an extremely complex network of physiological signals, conveying information regarding energy availability between the gastrointestinal (GI) tract and the central nervous system (CNS), driving motivation to either start or cease eating. The drive to eat is under strong influence of the CNS reward network. In recent years, functional magnetic resonance imaging (fMRI) studies have provided valuable insights into how food cues generate neural responses, which in turn determine eating behavior. For instance, increased food cue reactivity has been shown to predict greater food intake in the absence of hunger, a predisposition to weight gain, and attenuated weight loss from dietary interventions [5]. When intake continuously exceeds energy requirements, a chronically positive energy balance leads to weight gain and ultimately obesity.

Bariatric surgery is currently the most effective treatment for patients with severe obesity, leading to sustained weight loss. The most commonly bariatric procedures are the Roux-en-Y gastric bypass (RYGB) and the sleeve gastrectomy (SG) [6]. Observational studies and a number of randomized controlled studies suggest that RYGB and SG produce comparable weight loss and health improvements in the short-term [6]. Following RYGB and SG patients report reduced hunger in the fasted state, increased post-meal satiety, changes in taste, and altered food preferences away from energy-dense foods [7]. A reduced energy intake, as a result of this altered eating behavior, is the main driver for weight loss following bariatric surgery. Reduced food cue reactivity following bariatric surgery has been demonstrated in a number of fMRI studies, with reduced reward responses to energy-dense foods. Improving our understanding of food cue reactivity and how it reduces following bariatric surgery may pave the way for development of improved treatments for obesity.

A number of studies have investigated HH and food cue sensitivity using the Power of Food Scale (PFS), a reliable and validated questionnaire that measures an individual's hedonic appetite in well-nourished populations [8]. The PFS reflects a generalized tendency toward preoccupation with food and does not contain items describing actual food consumption or overeating [2, 9]. The PFS has satisfactory psychometric properties, including internal consistency and test–retest reliability with total scores not significantly affected by respondents’ hunger state [8]. Over 50 published studies have used the PFS to predict appetite-related outcomes including neural, cognitive, behavioral, anthropometric, and clinical measures [8]. People with obesity display markedly higher HH scores with the PFS compared to lean controls, while the relationship between PFS and BMI in the general population is less consistent [9].

A small number of studies have used the PFS scale to assess HH after bariatric surgery. In a retrospective study, Schultes et al*.,* found increased PFS scores in patients with obesity compared to normal weight subjects; however, PFS scores in patients more than 1 year post-RYGB were comparable to those seen in normal weight individuals [10]. A prospective study from the same group demonstrated marked reductions in PFS scores in patients with severe obesity after a mean of 15.9 months post-RYGB together with healthier dietary habits [11]. Finally, a longitudinal study measuring PFS scores in 16 adolescents with severe obesity at 3, 6, 12, 18, and 24 months post-RYGB demonstrated that reductions in HH paralleled reductions in BMI for the first 18 months. Interestingly, PFS scores rose mirroring modest increases in BMI at 24 months from surgery [12].

To date, only very limited data exist on the effect of SG, the most commonly undertaken bariatric procedure worldwide, on PFS scores [13]. Furthermore, existing data on PFS and bariatric surgery come from a limited number of studies with small patient numbers and longitudinal data are lacking. Moreover, none of the studies to date have investigated whether there is a relationship between HH changes over time and with total weight loss. There are no published studies reporting a direct comparison between the two most common bariatric procedures, the RYGB and SG, and PFS measures. The aim of our study was to longitudinally compare changes in PFS in a cohort of adult patients with severe obesity undergoing RYGB and SG and assess the correlation between the PFS scores and weight outcomes after surgery.

## Materials and methods

This study was designed as a prospective cohort study and was undertaken at Bariatric Centre for Weight Management and Metabolic Surgery, University College London Hospitals (UCLH) NHS Foundation Trust, London, UK. Inclusion criteria were adult patients (> 18 years), with a BMI ≥ 40 kg/m^2^ or BMI ≥ 35.0 kg/m^2^ with an obesity-related co-morbidity, who were planned to undergo an RYGB or SG as a primary bariatric procedure between October 2014 and December 2015 and were proficient in spoken and written English. Participation in this study was voluntary and informed consent was obtained in person by a healthcare professional. Ethical approval was obtained from the National Health Service Research Ethics Committee (ID#09/H0715/65) and the study was undertaken in accordance with the Helsinki Declaration.

Prior to surgery, all patients were assessed by a multidisciplinary team, including a bariatric surgeon, bariatric physician, bariatric specialist nurse, dietitian and psychologist, and their medical, surgical, and psychiatric history were assessed. Patients fulfilled the eligibility criteria outlined by the National Institute for Health and Care Excellence [14]. Following the initial assessment, a recommendation for surgical procedure was made by the multidisciplinary team, taking previous surgical, medical history, eating behavior, and informed patient preference into consideration, after standardized counseling including details, risks and benefits of each procedure. Post-surgery, patients were advised to follow a liquid diet for 2 weeks, followed by softer foods for a further 2 weeks, before resuming a solid diet thereafter. RYGB and SG were undertaken as previously described [15].

Weight was measured using a walkthrough platform by a trained healthcare professional. Participants completed the PFS which was generously provided to us by Michael Lowe, Department of Psychology, Drexel University, Philadelphia, before and approximately 6 months after bariatric surgery. The PFS contains 15 items reflecting an individual’s responsiveness to the food environment grouped into three domains according to food proximity: (1) food readily available in the environment but not physically present (food available = FA), (2) food present but not tasted (food present = FP), and (3) food when first tasted but not consumed (food tasted = FT). For each item, subjects were asked to score their reactions on a five-level scale ranging from 1 = “I do not agree at all” to 5 = ”I strongly agree”. The mean of the items comprising each of the three domain scores was calculated to obtain an aggregated score (total score = TS) (Table1) [2, 9].

**Table 1:** The power of food scale (pfs) items

1	I find myself thinking about food even when I am not physically hungry
2	I get more pleasure from eating than I do from almost anything else
3	If I see or smell a food I like, I get a powerful urge to have some
4	When I’m around fattening food I love, it’s hard to stop myself from at least tasting it
5	It’s scary to think of the power that food has over me
6	When I know a delicious food is available, I can’t help myself from thinking about having some
7	I love the taste of certain foods so much that I can’t avoid eating them even if they’re bad for me
8	Just before I taste a favorite food, I feel intense anticipation
9	When I eat delicious food I focus a lot on how good it tastes
10	Sometimes, when I’m doing everyday activities, I get an urge to eat ‘out of the blue’ (for no apparent reason)
11	I think I enjoy eating, a lot more than most other people
12	Hearing someone describe a great meal makes me really want to have something to eat
13	It seems like I have food on my mind a lot
14	It’s very important to me that the foods I eat are as delicious as possible
15	Before I eat a favorite food my mouth tends to flood with saliva
FA	Items: (1 + 2 + 5 + 10 + 11 + 13)/6
FP	Items: (3 + 4 + 6 + 7)/4
FT	Items: (8 + 9 + 12 + 14 + 15)/5
TS	Domains: (FA + FP + FT)/3

Table illustrating the 15 items of the PFS (reactions ranging from 1”I do not agree at all” to 5”I strongly agree”). Calculation of the PFS domains FA, FP, FT, and TS [2, 9]”

*FA* food available, *FP* food present, *FT* food tasted, *TS* total score

Clinical data including height and weight on the date of surgery were collected from the clinical records. Age was calculated as the difference between the date of birth and date of surgery. Baseline BMI was calculated from the weight measured in kg, divided by the square of height measured in meters on the day of surgery. Postoperative weight loss (WL) was determined relative to the weight on the day of surgery. Percentage WL (%WL) was chosen as the outcome measure for weight change, as %WL is less influenced by baseline BMI than % excess weight loss or BMI change.

Data were analyzed using GraphPad Prism version 8 and SPSS statistical software. Mean and standard error of mean (SEM) were calculated. Continuous data were assessed for normality using D'Agostino and Pearson omnibus normality test. Parametric or one-way analysis of variance (ANOVA) and nonparametric tests were used as appropriate. Chi-square tests were used for categorical data. Linear regression analyses were performed. Significance was assumed below the 0.05 level.

## Results

### Demographics

A total of 393 patients were invited to participate in the study at their bariatric surgery pre-assessment appointment and 175 completed the PFS pre-surgery. Out of these, 36 patients were subsequently excluded: 29 did not proceed to surgery, 3 did not attend post-operative follow-up, and 4 did not complete the follow-up PFS questionnaire. A total of 139 participants were included in the final analysis: 95 following SG and 44 post-RYGB. The study patient population demographics are illustrated in Table 2. The RYGB and SG groups were comparable in terms of age, BMI at surgery, time of post-op questionnaire completion, post-operative %WL, and incidence of T2D; however, the proportion of female participants was higher in the RYGB group.

**Table 2:** Participant characteristics

Age (years)		BMI at surgery (kg/m^2^)		Time from surgery (days)		%WL		Sex (%)		T2D (%)	
SG	RYGB	SG	RYGB	SG	RYGB	SG	RYGB	SG	RYGB	SG	RYGB
44.87 ± 1.12	49.02 ± 1.79	45.33 ± 0.79	44.44 ± 0.94	214.6 ± 6.41	220.2 ± 10.06	21.66 ± 0.67	22.76 ± 0.90	69.47% Female	86.36% Female	27.37%	38.64%
*p *= 0.052		*p* = 0.756		*p* = 0.537		*p* = 0.173		*p* = 0.033*		*p* = 0.181	

Table illustrating the patient characteristics among the two study cohorts. Age, Body Mass Index (BMI) at surgery, time post-surgery, percentage weight loss (%WL), sex, and type 2 diabetes (T2D) in patients following Roux-en-Y gastric bypass (RYGB) and sleeve gastrectomy (SG) (mean ± SD or %) *p value ≤0.05

### PFS scores

PFS scores were calculated for each of the questionnaire’s categories, FA, FP, FT, as well as the TS. Responses were compared between the RYGB and SG groups, prior to and after their primary bariatric procedure. At baseline, there were no differences in individual category or total PFS scores between the two groups, as illustrated in Table 3.

**Table 3:** Power of food scores at baseline

FA		FP		FT		Total	
SG	RYGB	SG	RYGB	SG	RYGB	SG	RYGB
2.49 ± 0.1	2.78 ± 0.16	2.91 ± 0.12	3.07 ± 0.18	2.30 ± 0.09	2.70 ± 1.32	2.68 ± 0.10	2.85 ± 0.14
*p* = 0.130		*p* = 0.553		*p* = 0.790		*p* = 0.337	

Comparison of scores among individual categories and total scores using the PFS in the RYGB and SG patients at baseline. Food Available (FA), Food Present (FP), Food tasted (FT), Roux-en-Y gastric bypass (RYGB), and sleeve gastrectomy (SG)

At 6 months post-surgery, a significant reduction was seen in total, FA, and FP scores in the RYGB group and in all categories in the SG group (Tables 4, 5). ANOVA analysis of variance demonstrated no differences between PFS subgroups post-RYGB; however, a difference was noted in the SG group between dFP and dFT with a significantly larger reduction in FP scores (ANOVA *p* = 0.0139*, significant difference between dFP and dFT).

**Table 4:** Post-RYGB: power of food scores post-RYGB

FA		FP		FT		Total	
Pre	Post	Pre	Post	Pre	Post	Pre	Post
2.78 ± 0.16	2.09 ± 0.14	3.07 ± 0.18	2.35 ± 0.17	2.70 ± 1.32	2.42 ± 0.13	2.85 ± 0.14	2.28 ± 0.13
*p* = 0.0006***		*p* = 0.0007***		*p* = 0.0983 (ns)		*p* = 0.0031**	

dFA	dFP	dFT	dTotal
− 0.71 ± 0.20	− 0.73 ± 0.23	− 0.27 ± 0.16	− 0.56 ± 0.18
ANOVA *p* = 0.3305 (ns)			

Comparison of scores among individual categories and total scores using the PFS in the RYGB patients at baseline and 6 months from surgery. Food Available (FA), Food Present (FP), Food tasted (FT), Roux-en-Y gastric bypass (RYGB), and sleeve gastrectomy (SG). (** indicates a p value ≤0.01 and  *** a p value ≤0.001)

**Table 5:** Post-SG: power of food scores post-SG

FA		FP		FT		Total	
Pre	Post	Pre	Post	Pre	Post	Pre	Post
2.49 ± 0.11	1.89 ± 0.09	2.91 ± 0.12	2.0 ± 0.09	2.65 ± 0.10	2.30 ± 0.09	2.67 ± 0.10	2.07 ± 0.08
*p* < 0.0001****		*p* < 0.0001****		*p* = 0.0001***		*p* < 0.0001****	

dFA	dFP	dFT	dTotal
− 0.61 ± 0.10	− 0.89 ± 0.12	− 0.35 ± 0.09	− 0.61 ± 0.09
ANOVA *p* = 0.0139* Significant difference between dFP and dFT			

Comparison of scores among individual categories and total scores using the PFS in the SG patients at baseline and 6 months from surgery. Food Available (FA), Food Present (FP), Food tasted (FT), Roux-en-Y gastric bypass (RYGB), and sleeve gastrectomy (SG) (** indicates a p value ≤0.01, *** a p value ≤0.001 and **** p value ≤0.0001

A direct comparison of delta scores in total and individual categories did not show any statistically significant differences in the magnitude of PFS score reductions between the two groups (SG vs RGB; dFA − 0.61 ± 0.10 vs − 0.71 ± 0.20, *p* = 0.618; dFP − 0.89 ± 0.12 vs − 0.73 ± 0.23, *p* = 0.499; dFT − 0.35 ± 0.09 vs − 0.27 ± 0.16, *p* = 0.824; dTS − 0.61 ± 0.09 vs − 0.56 ± 0.18, *p* = 0.792).

### Correlation of PFS change to %WL

The relationship between change in PFS scores compared to pre-surgery and post-operative %WL was examined. In the RYGB group, significant correlations were seen between %WL at 6 months and dFA (*R* − 0.2612 *p* = 0.0434*), dFP (*R* = − 0.2906 *p* = 0.0278*), and dTotal PFS score (*R* − 0.2867 *p* = 0.0296*). However, in the SG group, a significant correlation was observed between the dFP score and %WL (*R* = − 0.1981 *p* = 0.027*). dFA, dFT, and total dTotal PFS scores were not seen post-SG.

### Changes in PFS scores as predictors of post-operative %WL

Linear regression analyses were performed to investigate whether changes in PFS scores from baseline to 6 months predict post-operative %WL at 6, 12, and 24 months following RYGB and SG, respectively, using models correcting for sex and T2D status. Changes in delta PFS scores were not predictive of %WL at 6 months post-RYGB. However, dFA (*p *= 0.004**), dFP (*p* = 0.004**), and dTotal (*p* = 0.006**) scores at 6 months post-RYGB predicted 12 months %WL. The changes in PFS scores, however, did not predict %WL at 24 following RYGB. Changes in FT scores did not predict %WL at any time point following RYGB.

Models utilizing delta PFS scores form baseline to 6 months as predictors for %WL following SG interestingly showed a different pattern. At 6 months post-SG, dFA, dFP, dFT, and dTotal scores were not predictive of %WL. Delta PFS scores did also not predict %WL at either 6 or 12 months post-SG. However, in a model correcting for sex and T2D, dFP and dTotal PFS scores form baseline to 6 months post-surgery, were predictive of %WL at 24 months following SG, suggesting that these may have a role in sustaining weight loss achieved following SG in the longer term (Table 6).

**Table 6:** Linear regression models for %WL at 24 months post-SG

Model	B (unstandardized)	Standard error	Beta (standardized)	*p* value
dFP and %WL and 24 months post-SG				
Constant	25.455	3.795		
Sex	− 1.735	2.560	–0.790	0.500
Diabetes	− 4.680	2.577	–0.210	0.74
dFP	− 2.343	0.916	–0.298	0.013*
dTotal PFS score and %WL at 24 months post-SG				
Constant	26.341	3.718		
Sex	− 2.232	2.548	–0.102	0.384
Diabetes	− 5.107	2.609	–0.229	0.055
dTotal	− 3.059	1.252	–0.286	0.017*

Table illustrating the linear regression models evaluating the relationship between the change in the FP score (dFP) from baseline to 6 months and the 24-month post-operative percentage weight loss (top) and the relationship between the change in total PFS score (dTotal) and 24-month post-operative percentage weight loss (bottom). *p ≤0.05

## Discussion

In this prospective cohort study, we evaluated and compared the effect of RYGB and SG, the two most commonly performed bariatric procedures on various parameters of HH assessed by the PFS questionnaire. The PFS was developed as a quantitative measure of HH in 2009. Since then, over 50 published studies have used the PFS to evaluate this [8]. Here, we demonstrate that post-bariatric surgery changes in PFS scores can predict post-operative %WL in a procedure-related way.

Bariatric surgery engenders weight loss through a number of biological changes, which alter eating behavior and thereby result in a reduced energy intake, which is the main driver for sustained weight loss. Gut hormones, metabolically active polypeptides secreted along the GI tract in response to fasting and eating, act upon CNS centers involved in appetite regulation and generate either orexigenic or anorectic responses. Following bariatric surgery, gut hormone secretion profiles change as a result of the anatomical changes from the surgery. Altered gut hormone secretion profiles are thought to be key mediators for weight loss following RYGB and SG. RYGB results in a marked rise in meal-stimulated circulating levels of anorectic hormones peptide YY 36 (PYY) and glucagon-like peptide 1 (GLP-1); these changes are also seen post-SG but to a lesser extent. SG, in contrast, leads to a significant reduction in the orexigenic hormone ghrelin, by means of removing most of the ghrelin-producing cell population from the stomach. Ghrelin and PYY/GLP-1 act on appetite-regulating areas of the CNS in an opposing manner, stimulating orexigenic or anorectic responses, respectively [16].

Eating behavior is not only a key determinant of the pathogenesis of obesity but also of post-bariatric surgery weight loss. The changes in eating behavior seen following bariatric surgery have been shown to correlate with changes in GI physiology. Whereas the RYGB and SG are comparable in terms of their post-operative weight loss and changes in eating behavior, there are anatomically distinct procedures, and hence lead to differential changes upon GI physiology. Understanding the exact impact of the two procedures on both eating behavior and GI physiology therefore requires that these are studied in a comparative fashion. To date, a number of animal and human studies have demonstrated increased satiety associated with increased post-meal PYY and GLP-1 levels following RYGB and reductions in hunger correlating with reduced ghrelin levels post-SG [16]. A growing body of evidence from the fMRI literature also suggests that a reduced hedonic response to food cues is also directly related to post-RYGB increases in meal-stimulated GLP-1 and PYY secretion.

In this study, we utilized the PFS to directly compare the effects of RYGB and SG on HH, through assessing patients’ responses to all three sub-categories of the PFS, as well as the total scores. The two study cohorts were comparable at baseline with no significant differences in terms of their baseline characteristics nor their PFS scores at baseline. In both the RYGB and SG cohorts, significant reductions where seen at 6 months post-surgery in total PFS scores and subcategory scores with the exception of the dFT score post-RYGB. Although when directly comparing the delta values of total and individual category scores between the two cohorts of patients, no statistically significant differences were detected, and a larger reduction in HH hunger following SG was observed. The smaller numbers in the RYGB cohort potentially contribute to this not reaching statistical significance. However, given the fact that ghrelin-producing cells are removed in SG, it is biologically plausible that a greater post-surgical decrease in HH in the SG group could a consequence of more marked reductions in ghrelin levels, a hormone known to drive HH.

We demonstrated that at 6 months post-RYGB, changes in dFA and dTotal scores correlated with post-operative %WL. We subsequently developed linear regression models to investigate the role of HH reduction in predicting post-operative %WL following RYGB and SG. Following RYGB, the reduction in total PFS scores, as well as dFA and dFP scores at 6 months predicted %WL at 12 months from surgery. This relationship between the changes in PFS scores and %WL following RYGB could be explained by the overall increase in satiety and reduction in the hedonic value of food. These changes in eating behavior post-RYGB are believed to be largely driven by post-prandial rises in anorectic hormones, such as PYY, GLP-1, and OXM. It is well known that intravenous infusion of PYY modulates the response of corticolimbic and higher cortical brain areas to visual stimulation with food pictures [17]. Furthermore, RYGB can increase dopamine receptor density in reward-processing brain areas [18]. Modifications of the brain’s reward system after surgery, in specific brain structures involved in reward-processing and decision-making, have been detected by fMRI and PET scanning, using a selective radio ligand for the dopamine receptors [19].

Here, we demonstrate a distinct relationship between post-SG changes in HH and %WL, compared to RYGB. When correlating PFS delta scores to post-operative %WL following SG, a positive correlation with %WL was seen in the FP category only. Changes in PFS scores post-SG were not predictive of post-operative %WL at 6 months when the questionnaires where completed, or at 12 months.

Changes in dFP and dTotal scores at 6 months predicted post-operative %WL at 24 months from surgery. The findings following SG indicate that reduced responsiveness to the presence to food correlates with %WL and furthermore has predictive value in terms of longer term outcomes at 24 months from surgery. Food cues and the presence of food are known to trigger powerful physiological responses including an increase in circulating ghrelin, which in turn drive a desire to eat [20, 21]. Again, it is plausible that post-SG reductions in circulating ghrelin levels could lead to this reduction in responsiveness to the presence of food. Interestingly, reduction in HH post-SG is predictive of longer term weight loss outcomes. These findings further suggest that that patients with high HH scores and HH-driven eating behavior may particularly benefit from SG and the post-operative reductions in ghrelin. Pre-operative ghrelin measurement, combined with PFS evaluations, may therefore have a role in a precision-medicine driven approach to bariatric procedure selection.

Despite the successes of bariatric surgery in engendering weight loss, it is of note that up to 20% of patients either have a poor response or regain weight following bariatric surgery [22]. The rate of poor weight loss and weight regain is higher post-SG compared to RYGB [23, 24]. Our findings suggest that changes in HH could have predictive value as a tool to identify patients at risk of weight regain following SG. Further studies directly linking the eating behavior changes following bariatric surgery with the physiological changes than underpin them and investigating their relationship to long weight loss outcomes are now needed.

There are several strengths in this study. First, it is prospective. Second, it includes more than 100 patients undergoing either RYGB or SG with complete dataset up to 24 months post-surgery with all the date collected directly in the hospital with no self-reported measures. All questionnaires were collected during a clinical appointment and under the medical supervision of a healthcare professional. However, the limitations of this study also need to be considered. The smaller sample size in the RYGB group, a reflection SG now being the most commonly performed procedure, may have impacted upon our ability to detect certain differences, such as the lack of predictive value of reduction in scores at 6 months. In addition, our follow-up data were limited to 24 months post-surgery, which is not sufficient to capture the relationship between HH and weight regain. Furthermore, the lack of physiological data to accompany our HH evaluation using the PFS score, such as measurement of the gut hormone ghrelin, does not allow us to draw conclusions about the relationship between the changes we observed and post-operative reductions in ghrelin.

In conclusion, we demonstrated that reduction in HH, assessed with PFS questionnaires is associated with %WL in the short term and can predict weight loss outcomes in the longer term in a procedure-related way; at 12 months post-RYGB (dFA, dFP, and dTotal) and 24 months post-SG (dFP, dTotal). Our findings suggest a potential link between post-bariatric surgery changes in HH and post-operative weight loss, in line with previous research, but also support the fact that RYGB and SG alter eating behavior in a physiologically distinct manner. Larger longitudinal studies combining PFS with gut hormone analyses following bariatric surgery are warranted. However, our findings highlight that there may be a role PFS to identify people who may require additional support following bariatric surgery.
